# Evaluation of the effects of differences in silicone hardness on rat model of lumbar spinal stenosis

**DOI:** 10.1371/journal.pone.0251464

**Published:** 2021-05-13

**Authors:** Hyunseong Kim, Jin Young Hong, Wan-Jin Jeon, Junseon Lee, In-Hyuk Ha

**Affiliations:** Jaseng Spine and Joint Research Institute, Jaseng Medical Foundation, Seoul, Republic of Korea; University of Messina, ITALY

## Abstract

Lumbar spinal stenosis (LSS), one of the most commonly reported spinal disorders, can cause loss of sensation and dyskinesia. In currently used animal models of LSS, the spinal cord is covered entirely with a silicone sheet, or block-shaped silicone is inserted directly into the spinal canal after laminectomy. However, the effects of differences between these implant materials have not been studied. We assessed the degree of damage and locomotor function of an LSS model in Sprague-Dawley rats using silicone blocks of varying hardness (70, 80, and 90 kPa) implanted at the L4 level. In sham rats, the spinal cord remained intact; in LSS rats, the spinal cord was increasingly compressed by the mechanical pressure of the silicone blocks as hardness increased. Inflammatory cells were not evident in sham rats, but numerous inflammatory cells were observed around the implanted silicone block in LSS rats. CD68+ cell quantification revealed increases in the inflammatory response in a hardness-dependent manner in LSS rats. Compared with those in sham rats, proinflammatory cytokine levels were significantly elevated in a hardness-dependent manner, and locomotor function was significantly decreased, in LSS rats. Overall, this study showed that hardness could be used as an index to control the severity of nerve injury induced by silicone implants.

## Introduction

Lumbar spinal stenosis (LSS) is a degenerative disease that affects the lumbar region of the spinal cord. The narrowing of the spinal canal occurs in the spine of nearly every adult and progresses slowly with age. In LSS, the narrowing of the spinal canal exerts pressure on the spinal cord, causing inflammation and pathological radicular pain owing to the release of several proinflammatory cytokines. As the disease gradually advances, paresthesia gains increased severity and the patient eventually develops nerve damage that typically persists for life [1, 2]. Thus, LSS can cause the development of various lasting complications, such as impaired gait and daily living skills, neuralgia, and voiding dysfunction, and can cause pain in regions other than the spine, thereby markedly reducing the quality of life [3, 4]. Therefore, the establishment of suitable animal models of LSS is essential for developing effective treatments. Such models can be used to investigate factors contributing to the pathogenesis of stenosis, its effects on function, and its pathophysiological mechanisms, as well as to study the clinical effects and mechanisms of action of drugs currently used to treat stenosis. Thus, it is important to develop an animal model that closely reflects the clinical state, behavioral patterns, and physiological state of stenosis. In animal models of spinal stenosis, a sheet or block of silicone is inserted between the spinal cord and lumbar vertebrae to artificially narrow the spinal canal and impart pressure on the nerves. However, conventional procedures have provided only size information about silicone used to induce the LSS [5–11]. The silicone used in this process is not of standardized hardness, which can result in heterogeneous and imprecise animal models. As a result, research findings pertaining to candidate factors for treatment may be inaccurate and insufficiently objective. Therefore, it is extremely important to establish a standardized, precise animal model of LSS and evaluate its accuracy. Silicone rubber has been widely used in medicine because of its biocompatibility and viscoelasticity. Silicone is characterized by its excellent thermal stability, electrical insulation, hydrophobicity, non-volatility, and safety in the human body [12]. In addition to tubing, this material has several applications in orthopedic and reconstructive surgery [13], including prosthetic ears and breasts [14–16]. Previous studies suggested that silicone hardness is generally 100 kPa or lower, making it non-abrasive toward biological tissues [17, 18]. In the present study, we hypothesized that different hardness of silicone block can be used as an index to control the severity of the nerve injury and to a standardized method of creating a LSS model that allows control of disease severity. When determining the optimal range of silicone hardness, one of the most important criteria is the physiological range of tissues stiffness. Tissue stiffness varies depending on the function and location of the tissues, from less than 1 kPa for neuronal tissue to greater than 100 kPa in bone [19, 20]. And, previous studies reported that biomaterial hardness is desirable as equal to bone stiffness. If higher than the bone stiffness, then it can be penetrated in the bone [21]. In particular, the compression failure of silicone material was also be considered. Compression failure has been reported to occur in 35–70 kPa of a thick-walled silicone [22]. We finally chose the range of silicone harness from more than 70 kPa to less than 100 kPa. silicone blocks of varying hardness were implanted to induce LSS in rats, and histological, molecular, and functional changes were compared as a function of hardness. Using this approach, we developed animal models that consistently reproduced human LSS and indirectly investigated the extent of nerve injury based on the inflammatory response and behavioral changes. Our results provide guidance on using the hardness index to control the severity of nerve injury induced by silicone implants and accurately and uniformly induce LSS in rats. This method will be extremely useful for identifying the effects of therapeutic agents and candidate substances for treating stenosis.

## Materials and methods

### Characteristics of silicone blocks

**Fig 1:**
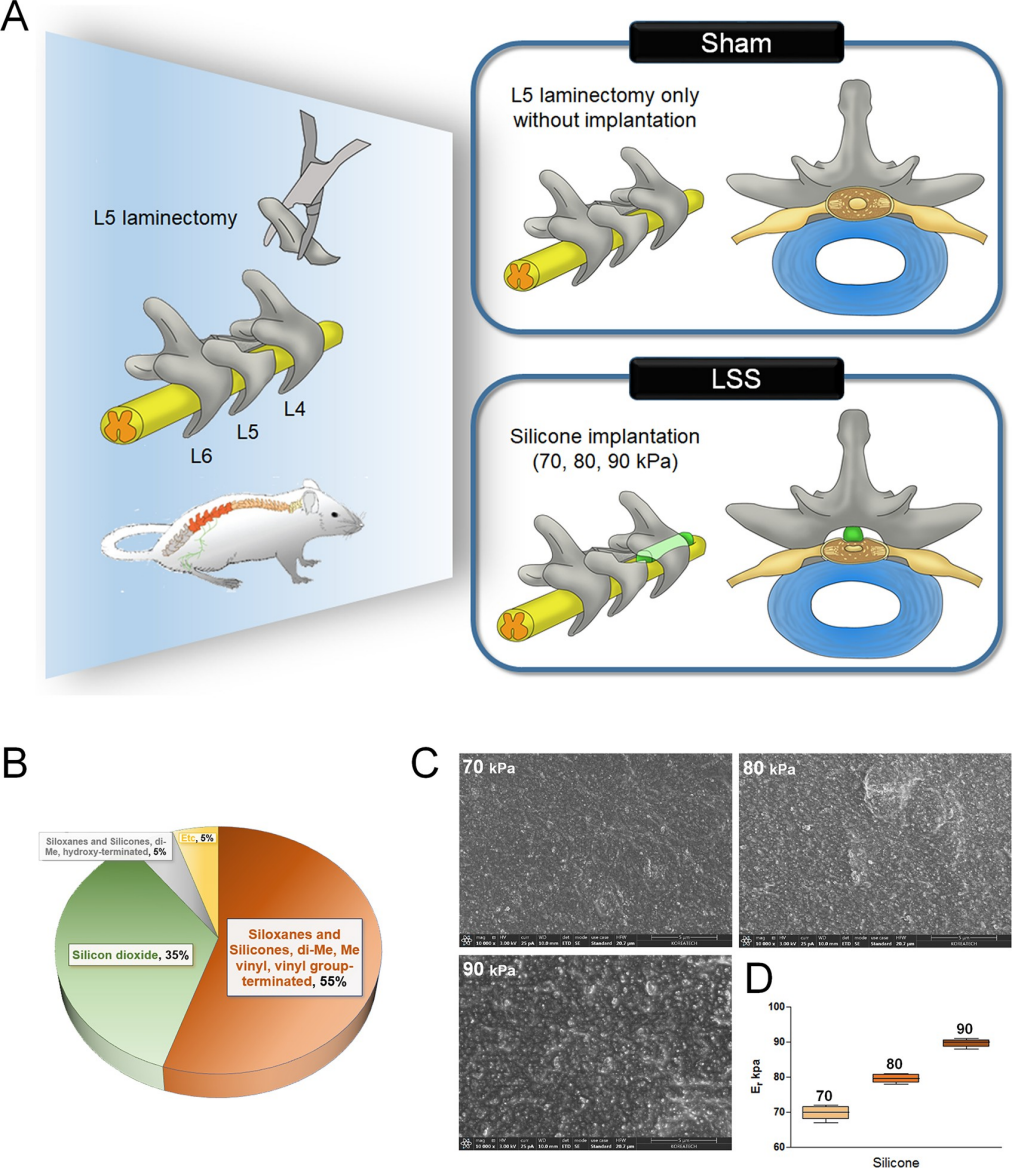
Characteristics of silicone blocks used for the induction of LSS. (A) Schematic of the experimental design for establishing a LSS model using silicone blocks with graded hardness. (B) Characterization of the silicone components. (C) SEM images of the surface morphology of silicone blocks with differing hardness. (D) Elastic modulus values from nanoindentation.

Silicone blocks (SH5180U) were obtained from KCC Corporation (Seoul, Korea). They contained siloxanes, silicones, di-Me, vinyl groups, and silicone dioxide. The mixing ratio of each compound to achieve the designated hardness is shown in Fig 1B. The surface morphology was examined by high-resolution scanning electron microscopy (FESEM, Helios NanoLab 600i FIB Workstation, FEI, Hillsboro, OR, USA). The elastic modulus was assessed to determine the hardness properties of silicone with a nanoindentation instrument (Hysitron, Minneapolis, MN, USA).

### Establishment of the rat spinal stenosis model

Male Sprague-Dawley rats (7 weeks of age, 230–250 g) were obtained from Daehan Bio Link (Chungju, Korea). All procedures were approved by the Jaseng Animal Care and Use Committee (JSR-2019-09-002-001). Rats were housed in separate cages under constant temperature (23–25°C) and humidity (45%–50%), and a 12-h light/dark cycle. Rats had free access to food and water. To establish the spinal stenosis model, rats were anesthetized with 2–3% isoflurane gas (Forane; BK Pham, Goyang, Korea) and a dorsal laminectomy was performed at L5 using fine rongeurs. The ligamentum flavum between the L4 and L5 was not removed. Lastly, a silicone block was inserted into the epidural space beneath the L4 with no. 5 fine forceps (JD-S-04; Jeung Do Bio & Plant Company, Seoul, Korea). Sham rats underwent laminectomy at the L5 level without an implant. The spinal cord was then covered with a Surgicel^®^ Absorbable Hemostat (Johnson and Johnson, Arlington, TX, USA). To prevent infection, all rats were injected intramuscularly with 40 mg/kg cefazolin sodium (Cefazolin Inj, Chong Kun Dang pharm, Korea) after suturing. All the rats were also administered an oral dose of 10 mg/kg acetaminophen syrup (Janssen Pharmaceuticals, Titusville, NJ, USA) for the pain management after the anesthesia had resolved until day 3 post-surgery. And, we monitored the daily health and behavior conditions of the animal until sacrifice following surgery. Further, we checked the signs associated with moderate-to-severe pain in the rat such as decreased activity or reluctance to move, decreased appetite, excessive licking or chewing of the surgery area, and spiky or rough-looking fur of the animal. Acetaminophen syrup at a dose of 10 mg/kg was orally administrated once daily for relief from the pain till the prevalence of associated signs. Rats were sacrificed 4 weeks after silicone implantation by carbon dioxide (CO_2_) inhalation. CO_2_ inhalation is a commonly used euthanasia method for small animals [23]. Each rat was carefully placed in a transparent acrylic chamber installed in an independent room separate from the animal room during euthanasia using CO_2_ inhalation. The CO_2_ gas was introduced at a 30% flow rate of cage volume per minute, adding to the appropriate air in the chamber to reach rapid unconsciousness with minimal distress to the animals. Rats were divided into 5 groups (n = 10/group): Sham group (Sham); no-silicone implantation, LSS group (70 kPa); 70 kPa silicone implantation, LSS group (80 kPa); 80 kPa silicone implantation, LSS group (90 kPa); 90 kPa silicone implantation. We have outlined our experimental procedures in more detail in a schematic diagram, which we have added in Fig 1A.

### Magnetic resonance imaging

Magnetic resonance imaging (MRI) was performed using the 9.4 T animal MRI scanner (Agilent 94/21; Agilent, Santa Clara, CA, USA) at the Korea Basic Science Institute in Ochang. Spectrometer electronics were equipped with a T/R-2/4 channels rat coil, and rat safety and comfort were monitored throughout the experiments. We obtained MRI sagittal image of the lumbar spine that had been implanted with the silicone block.

### Histological analyses

For hematoxylin and eosin (H&E) staining and immunohistochemistry, rats were perfused through the coronary artery with 0.9% normal saline (Sigma-Aldrich, St. Louis, MO, USA) and 4% paraformaldehyde (Biosesang, Seongnam, Korea) in 0.1 M phosphate-buffered saline (PBS) (pH 7.4). Spinal cord tissue at the L4 vertebra was collected and post-fixed overnight in 4% paraformaldehyde at 4°C. Samples were decalcified in Rapid Cal^TM^ (BBC Biochemical, Mt. Vernon, WA, USA) until complete decalcification. The samples were cryo-protected in 30% sucrose/PBS for 3 days and then embedded in cryo-blocks. Frozen tissues were cut on the axial plane into 16 μm-thick sections. H&E staining was performed at the implantation site to evaluate the extent of damage to the spinal cord caused by implantation of the silicone blocks at 4 weeks according to the standard protocol. Images of the stained sections were captured with an inverted microscope (Nikon, Tokyo, Japan). Quantification was performed by measuring the areas of the remaining spinal cord tissue using ImageJ software (v1.37, National Institutes of Health, Bethesda, MD, USA).

Luxol fast blue (LFB) staining was performed to evaluate demyelination at 4 weeks after induction of stenosis. Sections were washed in 0.1 M PBS, passed through 95% ethanol, and stained in LFB (Sigma-Aldrich) staining solution (1% LFB in 95% ethanol with 0.5% acetic acid) overnight at 60°C. The slides were rinsed with distilled water, differentiated in 0.05% lithium carbonate, and incubated in 70% ethanol until gray matter could be distinguished. The slides were dehydrated in an ethanol series, cleared with xylene, mounted in Vecta mounting medium (Vector Laboratories, Burlingame, CA, USA), and observed by microscopy (Nikon). We quantified the positive density and intensity of the LFB-stained myelin sheath using ImageJ software.

Immunohistochemistry analysis was performed on spinal cord sections to assess the inflammatory response in each group. The sections were incubated with 0.2% Triton X-100 in PBS for 5 min, rinsed twice with PBS for 5 min, and blocked with 2% normal goat serum (NGS) in PBS for 1 h. The following primary antibody, rabbit anti-monocyte/macrophage (1:500, Abcam, Cambridge, UK), were diluted in 2% NGS and incubated overnight at 4°C. After the sections were washed three times with PBS, they were incubated with an FITC-conjugated goat anti-rabbit IgG (Jackson ImmunoResearch Laboratories) at a dilution of 1:200 in 2% NGS in PBS. Following 2-h incubation, the sections were washed three times with PBS. The stained tissue sections were imaged using a confocal microscope (Eclipse C2 Plus, Nikon). The inflammatory response was quantified by manually counting the number of CD68^+^ cells and measuring the CD68^+^ intensity in highly magnified images at the implantation site using ImageJ software.

### Quantitative real-time polymerase chain reaction (qRT-PCR)

Changes in the mRNA levels of genes related to the inflammatory response after induction of stenosis were measured by qRT-PCR. Total RNA was isolated from the L4 spinal cord using an RNeasy Mini Kit (Qiagen, Hilden, Germany). cDNA was synthesized using random hexamer primers and AccuPower RT PreMix (Bioneer, Daejeon, Korea). Primers were designed using the UCSC Genome Bioinformatics and NCBI databases listed in Table 1. qRT-PCRs were performed in triplicate using the SYBR Green Supermix (Bio-Rad, Hercules, CA, USA) and the CFX Connect Real-Time PCR Detection System (Bio-Rad). Target gene expression was normalized to that of *β-actin* and has been expressed as the fold-change relative to the sham group.

**Table 1:** Primer sequences used for real-time PCR analysis.

**Gene**	**5′-3′**	**Primer sequence**
*iNOS*	Forward	ATGGCTTGCCCCTGGAAGTT
	Reverse	TGTTGGGCTGGGAATAGCAC
*COX2*	Forward	CTCAGCCATGCAGCAAATCC
	Reverse	GGGTGGGCTTCAGCAGTAAT
*TNF-α*	Forward	CCGACTACGTGCTCCTCACC
	Reverse	CTCCAAAGTAGACCTGCCCG
*IL-1β*	Forward	TTGCTTCCAAGCCCTTGACT
	Reverse	GGTCGTCATCATCCCACGAG
*IL-6*	Forward	CCACCCACAACAGACCAGTA
	Reverse	GGAACTCCAGAAGACCAGAGC
*IL-10*	Forward	TAACTGCACCCACTTCCCAG
	Reverse	AGGCTTGGCAACCCAAGTAA
*β-actin*	Forward	GCTACAGCTTCACCACCACA
	Reverse	GCCATCTCTTGCTCGAAGTC

### Enzyme-linked immunosorbent assays (ELISAs)

Expression levels of the proinflammatory markers interleukin (IL)-6 and tumor necrosis factor (TNF)-α in a 1-cm portion of each spinal cord, including the implantation site, were evaluated by ELISAs. Segments were homogenized in radioimmunoprecipitation assay (RIPA) buffer (GenDEPOT, Barker, TX, USA) containing a proteinase inhibitor (Millipore) using a Taco^TM^ Prep Bead Beater (GeneReach, Taichung, Taiwan) and centrifuged at 1000 rpm at 4°C for 3 min. Protein concentration was quantified using a bicinchoninic acid (BCA) protein assay kit (Thermo Fisher Scientific, Waltham, MA, USA). Supernatants were examined using ELISA kits (BD Biosciences, Franklin Lakes, NJ, USA) following the manufacturer’s instructions.

### Locomotor function assays

We used three tests to assess locomotor function after inducing stenosis: Von Frey test, Basso, Beattie, and Bresnahan (BBB) scale, and horizontal ladder test. The Von Frey test was used to measure sensitivity from a mechanical stimulus. The rats were adapted in clear acrylic cages on top of the wire mesh for 15 min before measuring their sensory thresholds. The calibrated Von Frey filaments were applied to the mid-plantar surface of both hind paws until a withdrawal response occurred using the Stoelting™ Ugo Basile electronic Von Frey instrument (Ugo Basile, Varese, Italy). The force applied to the animal’s hind paw was gradually increased from 5 g to 50 g. An avoidance response, including lifting, whipping, licking, or running off the foot during stimulation, was considered a positive reaction, and the response was automatically recorded. The average value of three or more measurements was used [24, 25]. The BBB scale is expressed as a score from 0 to 21 points (no hindlimb movement was scored 0 and normal hindlimb movement was scored 21) [26]. Two independent observers analyzed hindlimb motion in an open field (cylindrical acrylic box; 90 cm diameter, 15 cm high) for 4 min. The average value was used. The ladder walking test was used to test the ability of rats to maintain balance. All rats walked on a metal runway (2.5 cm between grids) from left to right three times, and their movements were captured with a digital camcorder. The score was calculated as follows: ladder score (%) = erroneous steps of hind limb/total steps of hind limb × 100 [27].

Locomotor functions were examined in each group every 7 days until sacrifice. All locomotor tests were recorded using a digital camera and performed by two observers who were blinded to the treatments.

### Statistical analyses

All numeric data are expressed as the means ± standard deviations. Prism 5 software (GraphPad, San Diego, CA, USA) was used for the analyses. One-way analysis of variance (ANOVA) with Tukey’s post-hoc test was used to confirm significant differences between the sham and LSS groups. Significance was considered at * p < 0.05, ** p < 0.01, or *** p < 0.001 vs. the sham group.

## Results

### Characteristics of silicone blocks used for the induction of LSS

Fig 1A shows the experimental design of this study. We used silicone blocks with a hardness of 70, 80, and 90 kPa to establish the LSS model. Rats in the sham group underwent only laminectomy without any implantation. The hardness of silicone was controlled by altering the mixing ratio of each compound (Fig 1B). The inset of the scanning electron microscopic image shows differences in the surface appearance of silicone of different hardness (Fig 1C). We confirmed the hardness of each silicone block by measuring its elastic modulus (Fig 1D). We accurately verified the morphological and individual features according to the hardness of the silicone, and the verified silicone was used to produce models of spinal stenosis.

### Surgical procedure and histological analysis of the LSS model

The inset of the surgical images illustrates the procedures performed (S1A Fig). Briefly, a midline incision was made on the back region through the skin, subcutaneous, and muscle layers. Next, the muscle around the lumbar spine was incised from L4 to L6, and a surgical window was secured using a retractor surgical tool. Laminectomy was performed at the L5 level, and silicone blocks of varying hardness were implanted at the L4 level. We evaluated the condition of the spinal cord following silicone block implantation by H&E staining. The shape of the spinal cord remained intact in sham-operated rats, whereas in the LSS rats, the spinal cord was compressed by the mechanical pressure exerted by the silicone block (S1B Fig). The T2-weighted sagittal plane magnetic resonance image also revealed the appearance of the silicone implant and compressed spinal cord (S1C Fig). The spinal cord showed a significantly decreased area because of compression after implantation of the silicone block, and the area gradually decreased with increasing silicone hardness (S1D Fig). These findings confirmed the importance of considering silicone hardness when inducing spinal stenosis, and the relationships between silicone hardness and spinal cord compression in the LSS model.

### Inflammation assessment of the LSS model

**Fig 2:**
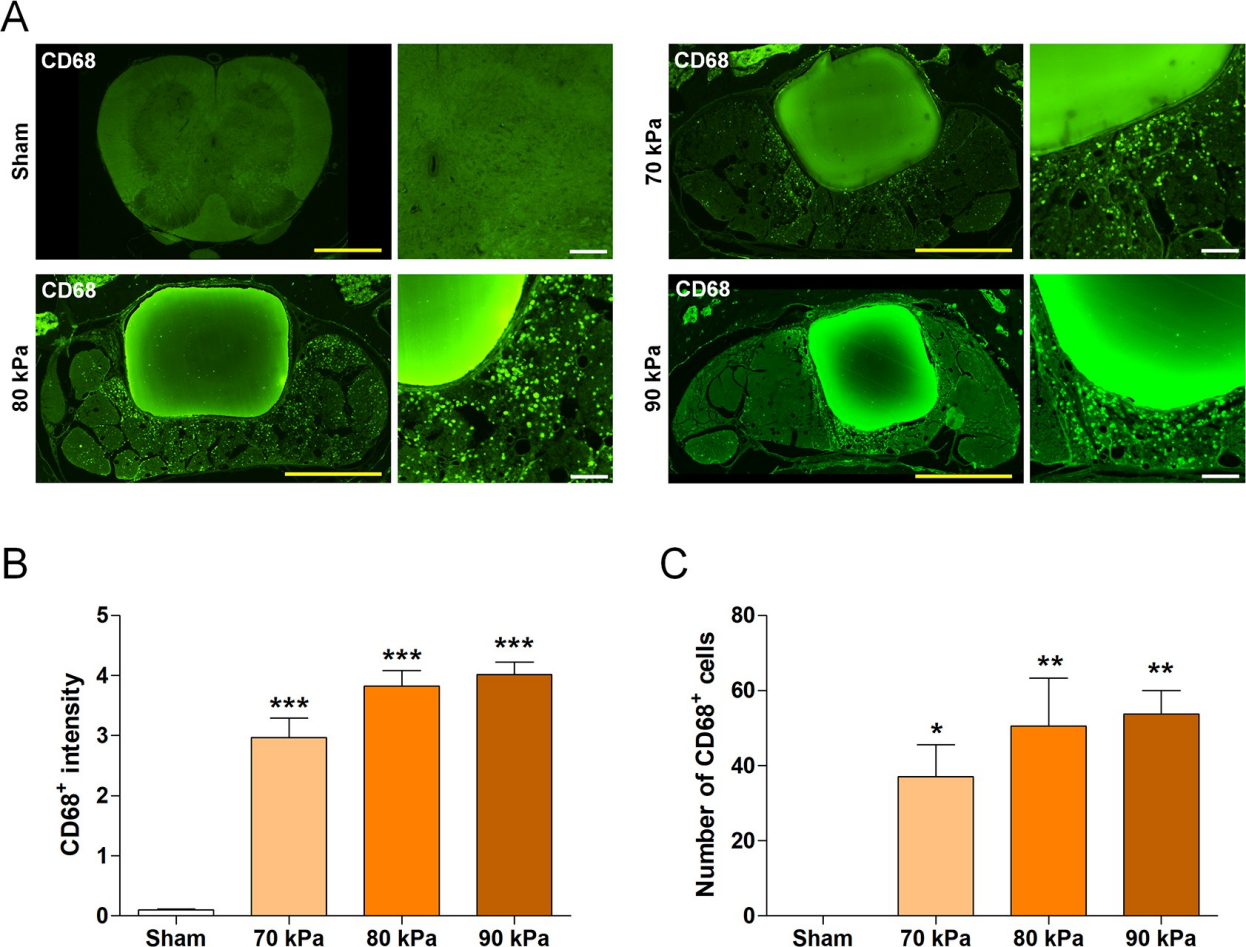
Inflammation assessment using the LSS model. (A) Representative immunofluorescence images of CD68^+^ macrophages in the spinal cord. Yellow scale bars = 1 mm and white scale bars = 100 μm. (B, C) Quantitative analyses of the (B) intensity and (C) number of CD68^+^ macrophages in the sham and LSS groups (n = 4 per group). Data are expressed as the means ± SEM. *P < 0.05, **P < 0.01, and ***P < 0.001 compared with the sham group analyzed by a one-way ANOVA with Tukey’s post-hoc test.

To evaluate the degree of inflammation as a function of silicone hardness, the numbers of inflammatory cells penetrating the implant region and extent of inflammatory marker expression were compared between the groups by immunohistochemistry. Inflammatory cells were not evident in sham-operated rats. In contrast, images of the LSS model sections revealed numerous inflammatory cells around the implanted silicone blocks (Fig 2A). We quantified the number of CD68^+^ cells and CD68^+^ intensity to analyze the extent of inflammation as a function of silicone hardness. The staining intensity and numbers of CD68^+^ cells were significantly higher in the silicone-implanted groups than in the sham group (Fig 2B and 2C). The mean CD68^+^ intensity and number were highest in the 90 kPa group, although the differences between silicone hardness were not significant. These results suggest that spinal stenosis induces a significant inflammatory response and that the degree of inflammation correlates with silicone hardness. Therefore, to produce a standardized and uniform stenosis model, it is important to implant silicone of precise hardness. This will enable accurate evaluation of the therapeutic effects of different substances.

### Demyelination assessment of the LSS model

**Fig 3:**
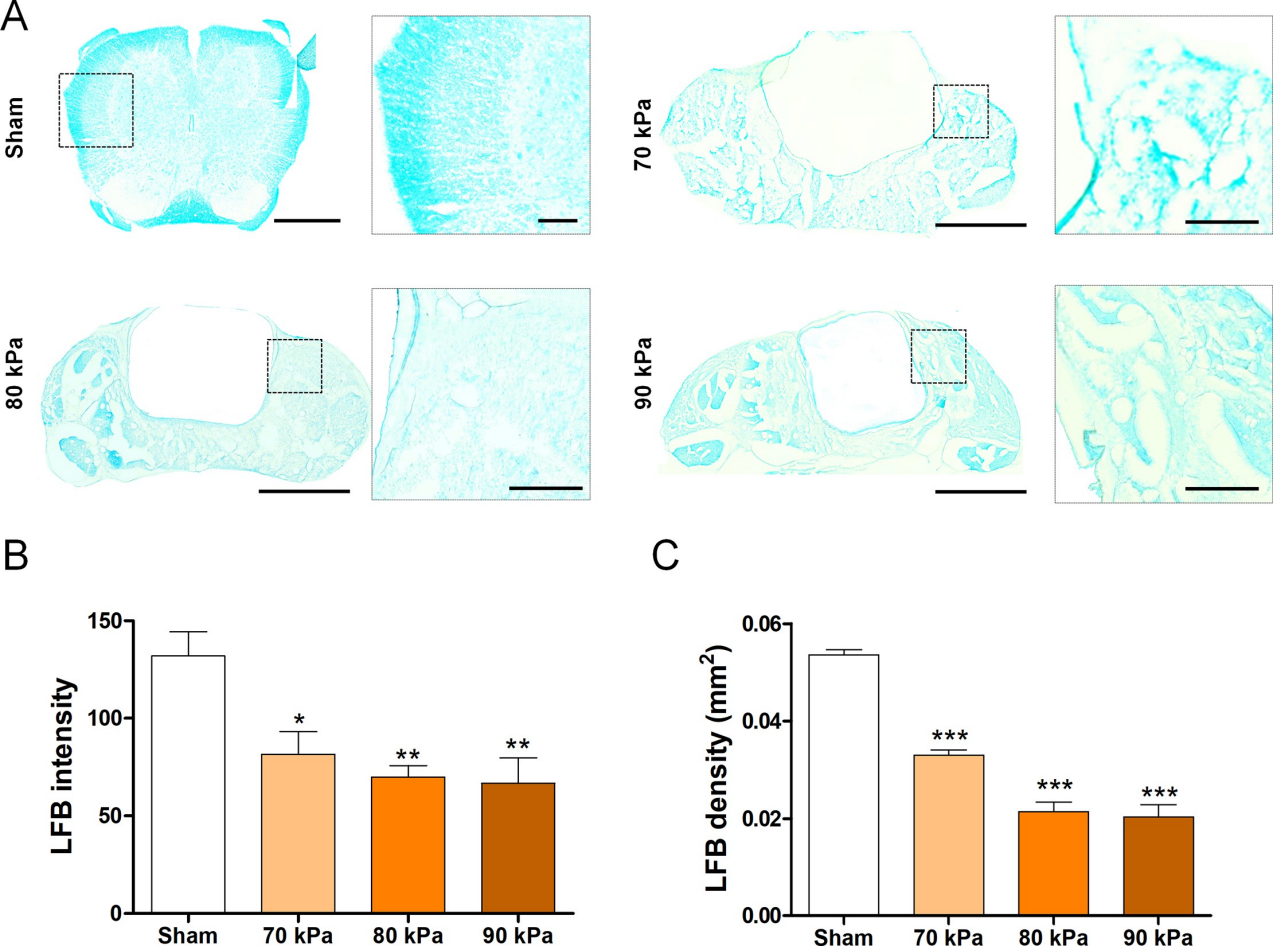
Demyelination assessment using the LSS model. (A) Representative LFB-stained images of the myelin sheath in the sham and LSS groups. Scale bars = 1 mm and 100 μm. (B, C) Quantitative analyses of (B) pixel intensity and (C) density in LFB-stained myelin sheaths (n = 4 per group). Data are expressed as the means ± SEM. *P < 0.05, **P < 0.01, and ***P < 0.001 compared with the sham group analyzed by a one-way ANOVA with Tukey’s post-hoc test.

The extent of demyelination was evaluated by LFB staining to check for damage to the myelin sheath after silicone implantation. Demyelination was increased after the implantation of silicone (Fig 3A). Quantitation of the density and intensity of the LFB stain within the spinal cord confirmed significantly increased demyelination in the silicone-implanted group (Fig 3B and 3C). Similar to the results obtained for the inflammatory response, although there was no significant difference between the silicone blocks in terms of hardness, the 90 kPa group showed the lowest average LFB+ intensity and area values. These results confirmed the presence of demyelination after implantation of silicone with varying hardness at the L4 level.

### Inflammation-related gene expression of the LSS model

**Fig 4:**
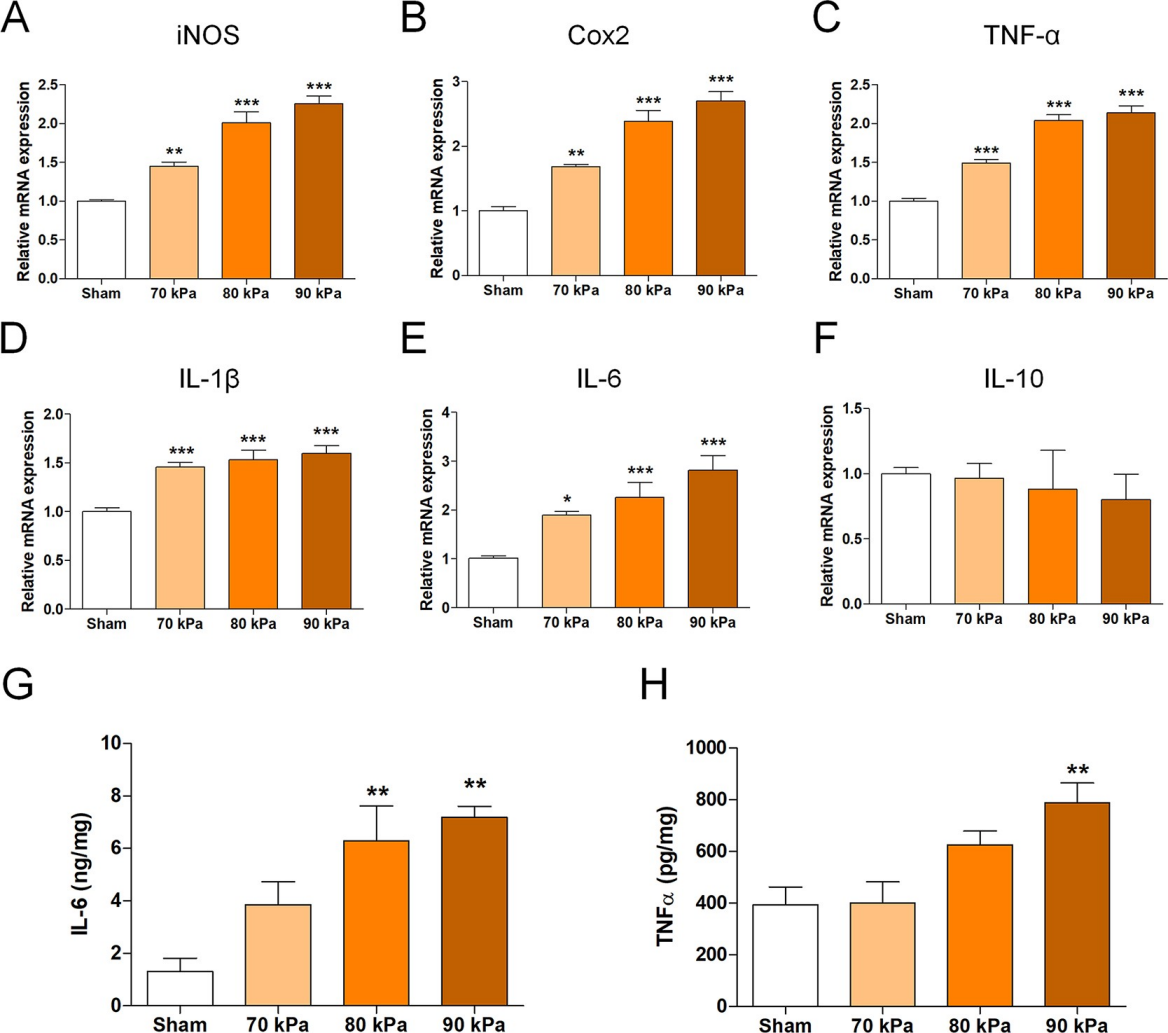
Inflammation-related gene expression in the LSS model. (A–F) Relative mRNA levels of pro- and anti-inflammatory enzymes and cytokines: (A) *iNOS*, (B) *Cox2*, (C) *TNF-α*, (D) *IL-1β*, (E) *IL-6*, and (F) *IL-10* in spinal cords implanted with a silicone block (n = 6 per group). (G, H) Enzyme-linked immunoassay results for inflammatory cytokine production. (G) IL-6, (H) TNF-α in each group 1 week after implantation of the silicone block (n = 6 per group). Data are expressed as the means ± SEM. *P < 0.05, **P < 0.01, and ***P < 0.001 compared with the sham group analyzed by a one-way ANOVA with Tukey’s post-hoc test.

We analyzed the mRNA levels of pro- and anti-inflammatory cytokine genes according to the silicone hardness by qRT-PCR. The mRNA levels of inducible nitric oxide synthase (*iNOS*), cyclooxygenase 2 (*Cox2*), *TNF-α*, *IL-1β*, and *IL-6*, which are representative cytokines that cause inflammation, were significantly elevated in the LSS groups compared with the sham group (Fig 4A–4E). However, the anti-inflammatory cytokine *IL-10* did not significantly differ between the LSS and sham groups (Fig 4F). Therefore, the inflammatory response dramatically increased after implantation of silicone. Similarly, at the protein level according to ELISAs, IL-6 and TNF-α levels were significantly higher in the LSS groups than in the sham group (Fig 4G and 4H). These data indicate that when the spinal cord or nerve roots are compressed because of the presence of implanted silicone, inflammation occurs as a result of spinal stenosis.

### Functional assessments of the LSS model

**Fig 5:**
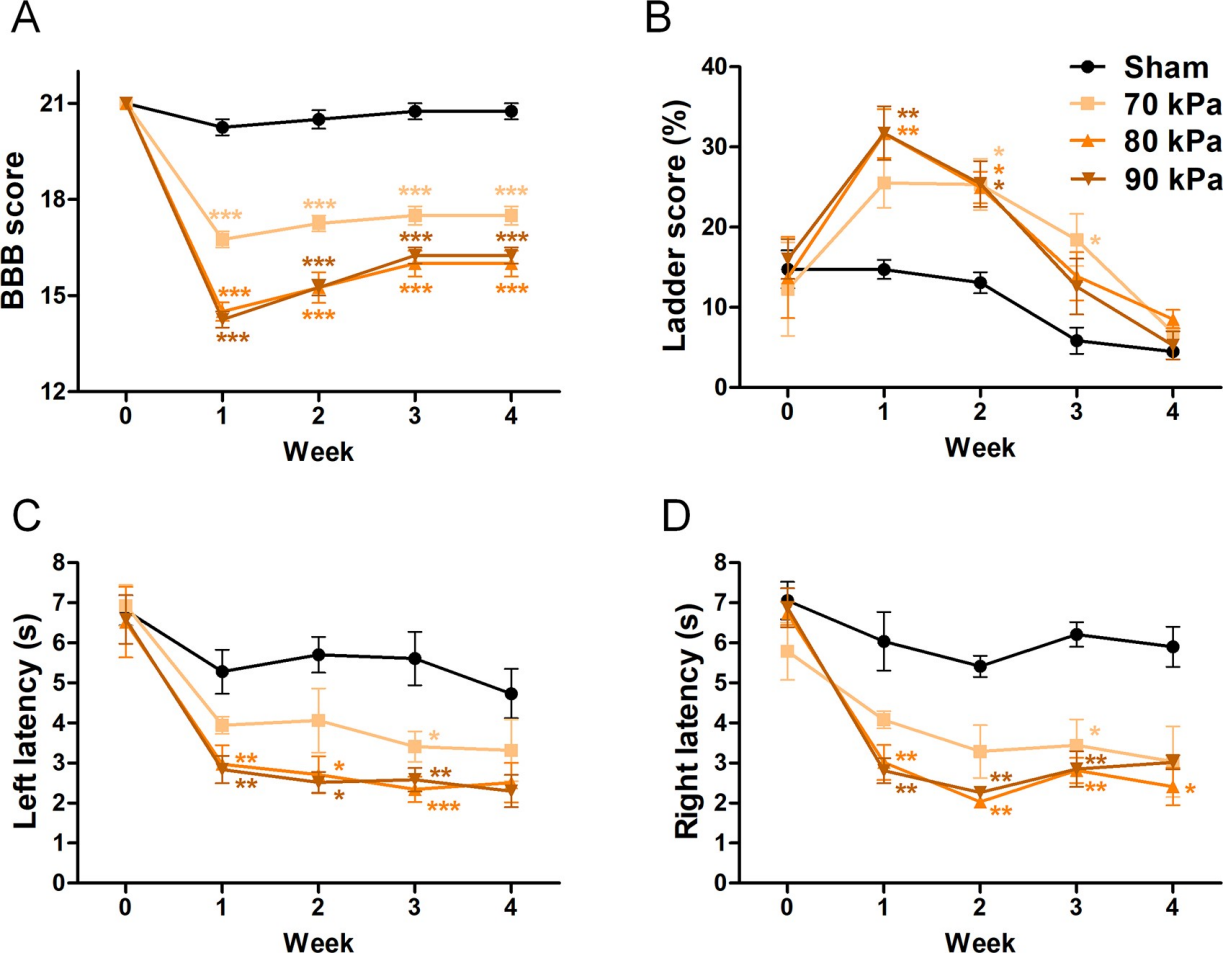
Functional assessment of the LSS model. (A) Basso, Beattie, and Bresnahan (BBB) score, (B) ladder score, and (C, D) Von Frey test (n = 6 per group). Data are expressed as the means ± SEM. *P < 0.05, **P < 0.01, and ***P < 0.001 compared with the sham group analyzed by a one-way ANOVA with Tukey’s post-hoc test.

The locomotor functions of LSS rats were assessed using three methods to reveal whether silicone implantation affected their behavior and motor functions: BBB, ladder, and von Frey tests. The BBB score was significantly decreased after 1 week in the silicone-implanted groups compared with that in the sham group. Rats implanted with 70 kPa silicone had an average BBB score of 16 points in week 1, which was approximately two points higher than those in rats implanted with 80 and 90 kPa silicone. However, the BBB scores showed no difference between 80 and 90 kPa silicone. Spontaneous behavioral recovery was observed during the first 2 weeks, whereas by 3 weeks, recovery ceased and the scores remained the same over time in all groups (Fig 5A). The ladder test revealed even more rapid and visible behavioral differences than the BBB test. Specifically, the LSS groups showed a lower frequency of stepping through forelimb-hindlimb coordination than the sham group. The sham group had a foot fault rate of approximately 15% at 1 week but showed adaptation in subsequent weekly assessments, with a foot fault rate of approximately 5% in the final assessment at 4 weeks. The 80 and 90 kPa silicone groups, but not the 70 kPa silicone group, showed a significantly increased foot fault frequency compared with the sham group up to 2 weeks. However, from 3 weeks, all LSS groups showed results similar to the sham group, indicating spontaneous recovery (Fig 5B). Although, these results do not elucidate whether harder silicone affects motor functions more severely, these data confirm that the movements of the rats were significantly impaired in the LSS models compared with the sham model. Moreover, rats in the 80 and 90 kPa groups showed impaired motor function compared with those in the 70 kPa group. Additionally, we performed the von Frey test to assess the mechanical sensory thresholds of the hind paw. Non-operated normal rats showed a withdrawal latency of 6–7 s. The pilot test revealed no difference between the rats of normal and sham groups (S2 Fig). Following stenosis-inducing surgery, rats showed increased sensitivity of the nerves in their legs, resulting in shorter withdrawal latencies. The 80 and 90 kPa groups showed faster withdrawal latencies than the 70 kPa group (Fig 5C and 5D). Therefore, a hypersensitivity response in the LSS model compared with the sham model was observed throughout the test period.

## Discussion

Animal models are essential for studying the pathological mechanisms of human diseases and investigating the mechanisms and clinical effects of current and novel drugs. We evaluated the histological and molecular changes associated with implantation of silicone blocks with varying hardness levels to establish a standardized, homogeneous rat model of LSS that mimicked similar injuries in humans. It has been difficult to develop an animal model in which the clinical course of the disease and histological findings accurately represent those observed in humans. However, preclinical trials are essential for precise assessments in clinical trials and the development of new treatment methods for human diseases. These models provide basic principles for developing optimal treatment methods. In many studies, a block or sheet of silicone is implanted into the lumbar spinal canal to produce an animal model of LSS; however, a precise, standardized model is currently lacking. Particularly, although biomaterials are used, no studies have evaluated the precise physical properties and hardness of the materials used to create models of LSS. Using scanning electron microscopy, we verified the differences in the surface appearance of silicone of different hardness. We predicted that as hardness increased, the spinal cord would be subjected to greater pressure and the injury would be more severe. Unexpectedly, there were no behavioral differences between the 80 and 90 kPa silicone implant groups. However, animals implanted with 70 kPa silicone showed a lower inflammatory response and faster spontaneous recovery of hindlimb locomotion than those implanted with 80 and 90 kPa silicone. In the 90 kPa hardness group, silicone was implanted into the lumbar spinal canal of the L4 vertebra. However, even with the implant positioned at the center, the silicone moved in tandem with the constant movement of the rat. This made it difficult to fix the silicone as compared with silicone of lower hardness (<90 kPa). Specifically, because the width of the spinal cord at the L4 vertebra is approximately 4 mm and width of the silicone block used in this study was 1 mm, despite the central implantation of silicone in the lumbar spinal canal, continuous movement of the silicone intensified with increasing hardness, resulting in increased functional variations between animals. Although the size of the silicone block could be adjusted to be equal to the width of the spinal cord, to limit the variables, we used the same size of silicone used in previous studies. This enabled us to examine changes specific to differences in silicone hardness. It was difficult to determine the efficacy and effects of long-term drug administration in this animal model of LSS because the 70 kPa hardness group showed fast and spontaneous behavioral recovery. In the 80 and 90 kPa groups, the accurate and uniform reproduction of LSS was possible, and a similar degree of nerve injury was observed in the histological analysis. These results demonstrate that hardness is a useful index for controlling the severity of nerve injury induced by a silicone implant. Presently, patients with LSS are graded clinically according to severity, and the treatment strategies and responses differ depending on the grade. To accurately assess the effects of different strategies as a function of disease severity, it is essential to systematically compare the degrees of reproduction of clinical symptoms, injury severity, and treatment outcomes using models with differing characteristics. Various approaches have been used to study treatments for LSS. Although complete recovery of patients has not been achieved, different treatment methods have been suggested or attempted based on pathophysiological studies, with numerous studies ongoing. Experimental animal models that can perfectly reproduce the various symptoms and abnormal findings during recovery observed in human patients with LSS are currently lacking. However, it is important to continue conducting research to select animal models suited to these research objectives to determine the strengths and weaknesses of each method. Additionally, studies are warranted for the development of new animal models that will enable more precise investigation of the pathogenesis and pathophysiology of LSS.

## Conclusions

There are differences in the clinical treatment of LSS depending on its severity. Therefore, the selection of an appropriate animal model with different severities is essential to increase the accuracy of preclinical results. Hardness is a useful index for controlling the severity of LSS induced by silicone implants. Our results show for the first time that the range of the silicone block hardness can provide a reference for making the LSS model mild, moderate, or severe in rats. Thus, the hardness control will enable the development of models, accurately reproducing the clinical symptoms, injury severity, and treatment outcomes observed in human LSS.

## Supporting information

S1 Fig Surgical procedure and histological analysis of the LSS model. (A) Surgical procedures of the LSS model using silicone blocks with different hardness. (B) Representative images of the H&E-stained sections of each group four weeks after implanting silicone blocks with differing hardness. Scale bars = 1 mm. (C) Sagittal T2 magnetic resonance image showing implantation of a silicone block at the L4 level. (D) Quantification of the compressed area of the spinal cord in each group (n = 4 per group). Data are expressed as the means ± SEM. ***P < 0.001 compared with the sham group analyzed by a one-way ANOVA with Tukey’s post-hoc test.
(TIF)

S2 Fig A pilot test result of an Von Frey assay between the rats of normal and sham groups. (TIF)

S1 Video Video protocol for surgical procedure of the LSS model. (WMV)

S1 File (DOCX)
