# Supplementary figures and images for: Evaluation of the effects of differences in silicone hardness on rat model of lumbar spinal stenosis
Source: PLoS One. 2021 May 13;16(5):e0251464. doi: 10.1371/journal.pone.0251464 (PMC8118556; doi:10.1371/journal.pone.0251464)

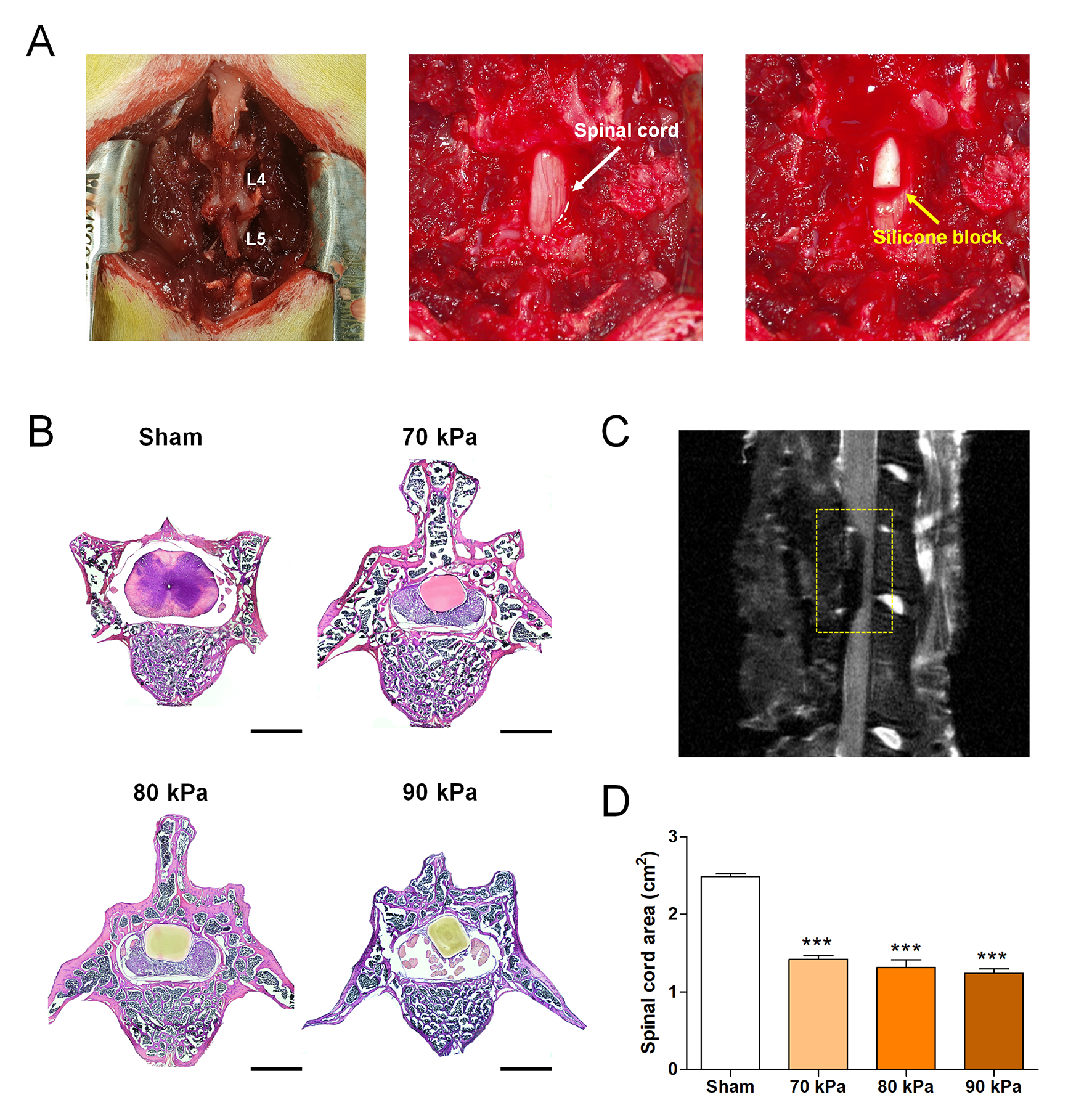

Supplement: S1 Fig — (A) Surgical procedures of the LSS model using silicone blocks with different hardness. (B) Representative images of the H&E-stained sections of each group four weeks after implanting silicone blocks with differing hardness. Scale bars = 1 mm. (C) Sagittal T2 magnetic resonance image showing implantation of a silicone block at the L4 level. (D) Quantification of the compressed area of the spinal cord in each group (n = 4 per group). Data are expressed as the means ± SEM. ***P < 0.001 compared with the sham group analyzed by a one-way ANOVA with Tukey’s post-hoc test. (TIF) [file pone.0251464.s001.tif]

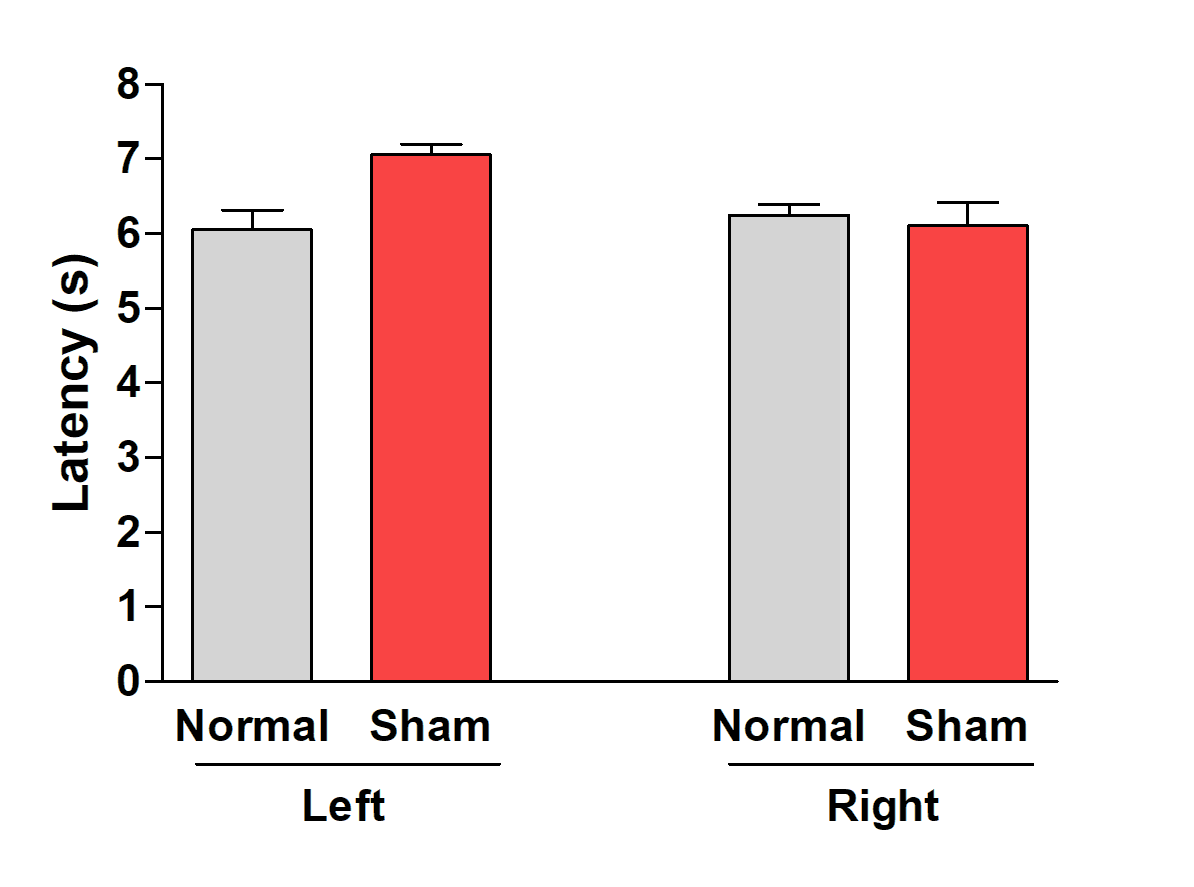

Supplement: S2 Fig — (TIF) [file pone.0251464.s002.tif]
