# Supplementary material for: Evaluation of the effects of differences in silicone hardness on rat model of lumbar spinal stenosis
Source: PLoS One. 2021 May 13;16(5):e0251464. doi: 10.1371/journal.pone.0251464 (PMC8118556; doi:10.1371/journal.pone.0251464)
Supplement: S1 File — (DOCX) [file pone.0251464.s004.docx]

Article title: Evaluation of the effects of differences in silicone hardness on rat model of lumbar spinal stenosis (PONE-D-21-00177)

Authors: Hyunseong Kim, Jin Young Hong, Wan-Jin Jeon, Junseon Lee, In-Hyuk Ha

The following Supporting information is available for this article:

**Intensity measurement using ImageJ software (v1.37)**

1. Background subtraction for intensity analysis

Process → Subtract background

1. Analyze → Measure, to measure signal intensity

**Counting of total ED1 positive cells**

1. Open the image → Manually cell counting


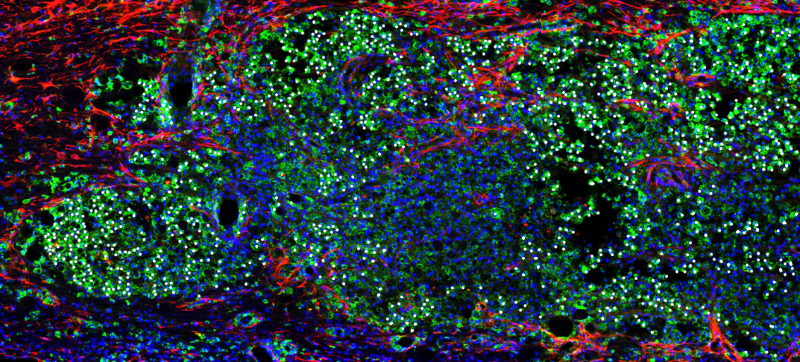


1.
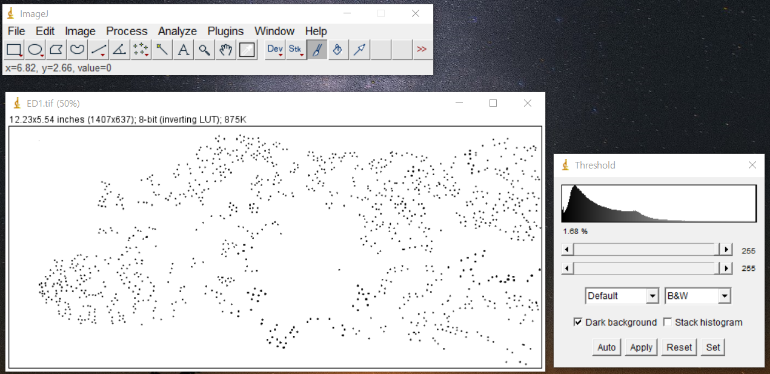
Image → 8 bit → Adujst → Threshold → Apply
2.
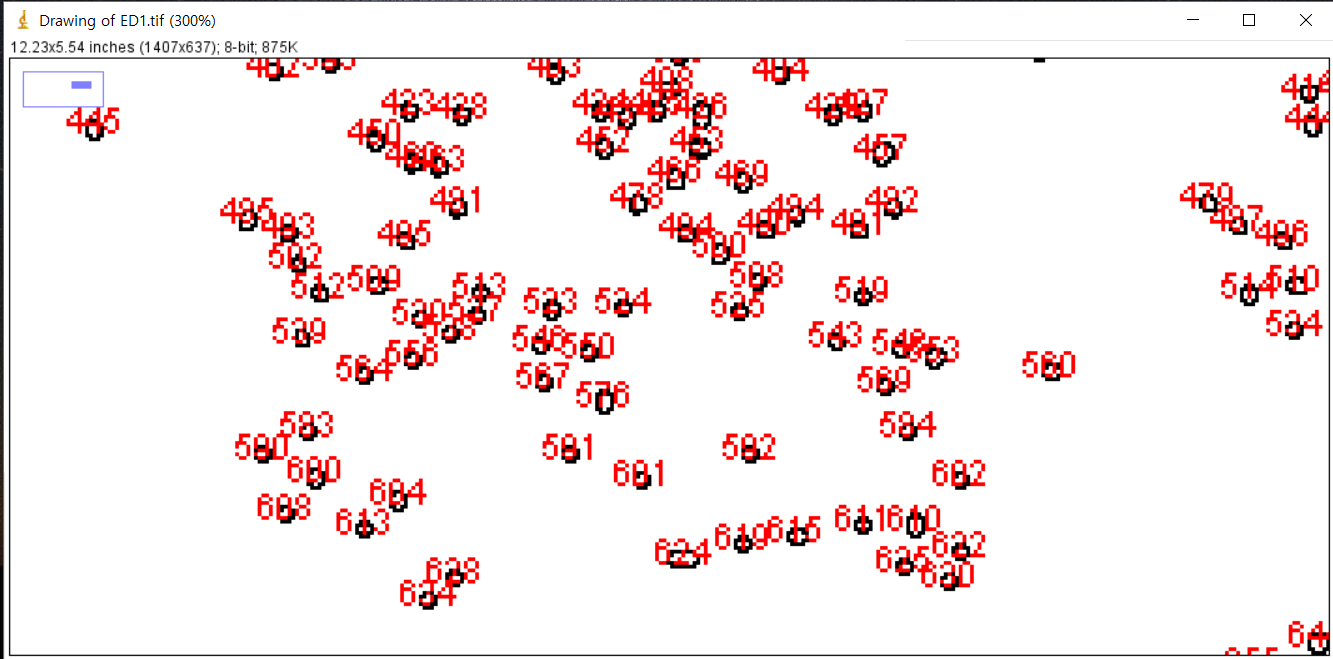
Analyze → Analyze particles → Count


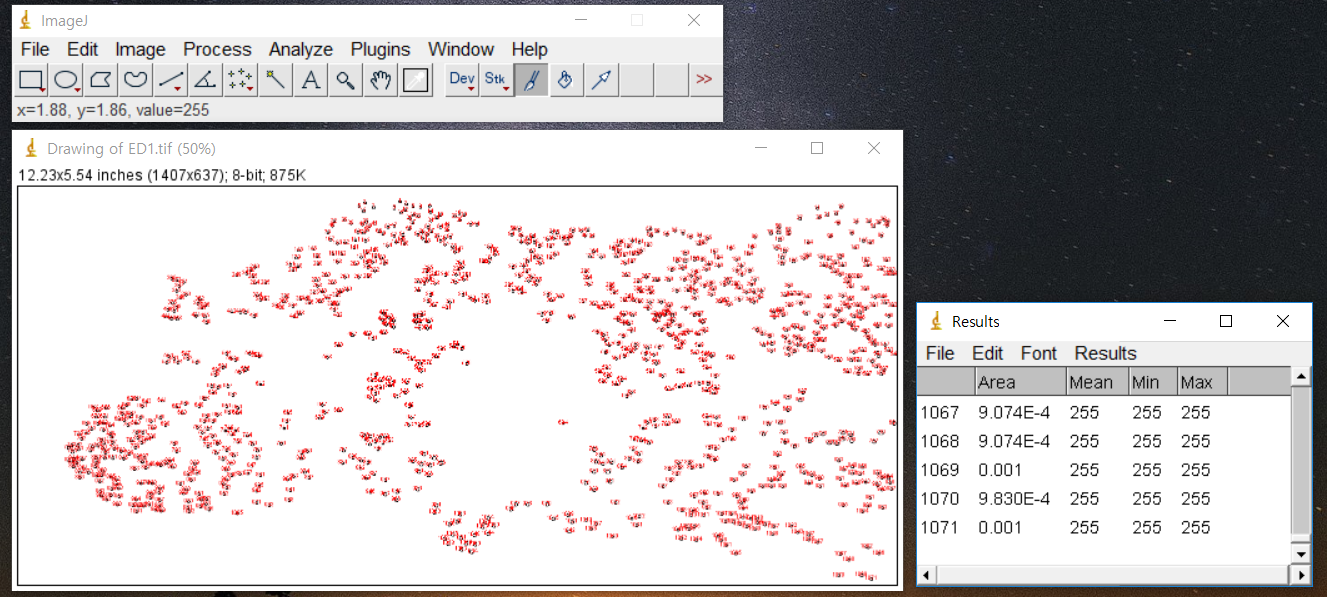


**S1 Table. The values used to build Fig 2B, C graph**

| **Fig 2B. CD68 intensity** | | | | | | | | | | |
| --- | --- | --- | --- | --- | --- | --- | --- | --- | --- | --- |
| **Test details** | **Mean 1** | | **Mean 2** | | **Mean Diff.** | | **95.00% CI of diff.** | | **Adjusted P Value** | |
| Sham vs. 70 kPa | 0.1028 | | 2.968 | | -2.865 | | -3.792 to -1.937 | | <0.0001 | |
| Sham vs. 80 kPa | 0.1028 | | 3.824 | | -3.721 | | -4.648 to -2.793 | | <0.0001 | |
| Sham vs. 90 kPa | 0.1028 | | 4.017 | | -3.914 | | -4.841 to -2.987 | | <0.0001 | |
| **Fig 2C. Number of CD68+ cells** | | | | | | | | | | |
| **Test details** | | **Mean 1** | | **Mean 2** | | **Mean Diff.** | | **95.00% CI of diff.** | | **Adjusted P Value** |
| Sham vs. 70 kPa | | 0 | | 37 | | -37 | | -69.27 to -4.727 | | 0.0227 |
| Sham vs. 80 kPa | | 0 | | 50.57 | | -50.57 | | -81.67 to -19.47 | | 0.0014 |
| Sham vs. 90 kPa | | 0 | | 53.67 | | -53.67 | | -85.94 to -21.39 | | 0.0011 |

**S2 Table. The values used to build Fig 3B, C graph**

| **Fig 3B. LFB intensity** | | | | | | | | | |
| --- | --- | --- | --- | --- | --- | --- | --- | --- | --- |
| **Test details** | | **Mean 1** | | **Mean 2** | **Mean Diff.** | | **95.00% CI of diff.** | **Adjusted P Value** | |
| Sham vs. 70 kPa | | 131.9 | | 81.51 | 50.43 | | 11.01 to 89.85 | 0.0107 | |
| Sham vs. 80 kPa | | 131.9 | | 70 | 61.94 | | 23.96 to 99.93 | 0.0014 | |
| Sham vs. 90 kPa | | 131.9 | | 66.71 | 65.24 | | 25.81 to 104.7 | 0.0012 | |
| **Fig 3C. LFB density** | | | | | | | | | |
| **Test details** | **Mean 1** | | **Mean 2** | | **Mean Diff.** | **95.00% CI of diff.** | | | **Adjusted P Value** |
| Sham vs. 70 kPa | 0.05358 | | 0.03302 | | 0.02056 | 0.01414 to 0.02699 | | | <0.0001 |
| Sham vs. 80 kPa | 0.05358 | | 0.02139 | | 0.03219 | 0.02576 to 0.03861 | | | <0.0001 |
| Sham vs. 90 kPa | 0.05358 | | 0.02026 | | 0.03332 | 0.02690 to 0.03975 | | | <0.0001 |

**S3 Table. The values used to build Fig 4A-F graph**

| **Fig 4A-F. Real-time PCR** | | | | | |
| --- | --- | --- | --- | --- | --- |
| **INOS** | **Mean 1** | **Mean 2** | **Mean Diff.** | **95.00% CI of diff.** | **Adjusted P Value** |
| Sham vs. 70 kPa | 1 | 1.452 | -0.4522 | -0.7862 to -0.1182 | 0.0046 |
| Sham vs. 80 kPa | 1 | 2.011 | -1.011 | -1.345 to -0.6771 | <0.0001 |
| Sham vs. 90 kPa | 1 | 2.257 | -1.257 | -1.591 to -0.9226 | <0.0001 |
| **COX-2** | **Mean 1** | **Mean 2** | **Mean Diff.** | **95.00% CI of diff.** | **Adjusted P Value** |
| Sham vs. 70 kPa | 1.005 | 1.685 | -0.6797 | -1.130 to -0.2291 | 0.0016 |
| Sham vs. 80 kPa | 1.005 | 2.39 | -1.384 | -1.835 to -0.9339 | <0.0001 |
| Sham vs. 90 kPa | 1.005 | 2.702 | -1.697 | -2.118 to -1.275 | <0.0001 |
| **TNF-α** | **Mean 1** | **Mean 2** | **Mean Diff.** | **95.00% CI of diff.** | **Adjusted P Value** |
| Sham vs. 70 kPa | 1 | 1.491 | -0.4905 | -0.7432 to -0.2379 | <0.0001 |
| Sham vs. 80 kPa | 1 | 2.043 | -1.043 | -1.295 to -0.7900 | <0.0001 |
| Sham vs. 90 kPa | 1 | 2.141 | -1.141 | -1.393 to -0.8880 | <0.0001 |
| **IL-1β** | **Mean 1** | **Mean 2** | **Mean Diff.** | **95.00% CI of diff.** | **Adjusted P Value** |
| Sham vs. 70 kPa | 1 | 1.455 | -0.4554 | -0.7178 to -0.1930 | 0.0002 |
| Sham vs. 80 kPa | 1 | 1.534 | -0.5336 | -0.7897 to -0.2775 | <0.0001 |
| Sham vs. 90 kPa | 1 | 1.598 | -0.5976 | -0.8675 to -0.3276 | <0.0001 |
| **IL-6** | **Mean 1** | **Mean 2** | **Mean Diff.** | **95.00% CI of diff.** | **Adjusted P Value** |
| Sham vs. 70 kPa | 1.012 | 1.894 | -0.8818 | -1.606 to -0.1573 | 0.0134 |
| Sham vs. 80 kPa | 1.012 | 2.263 | -1.251 | -1.976 to -0.5266 | 0.0004 |
| Sham vs. 90 kPa | 1.012 | 2.816 | -1.804 | -2.544 to -1.065 | <0.0001 |
| **IL-10** | **Mean 1** | **Mean 2** | **Mean Diff.** | **95.00% CI of diff.** | **Adjusted P Value** |
| Sham vs. 70 kPa | 0.9999 | 0.9641 | 0.03577 | -0.1926 to 0.2641 | 0.9618 |
| Sham vs. 80 kPa | 0.9999 | 0.8812 | 0.1187 | -0.1177 to 0.3550 | 0.4678 |
| Sham vs. 90 kPa | 0.9999 | 0.8012 | 0.1987 | -0.02966 to 0.4270 | 0.0985 |

**S4 Table. The values used to build Fig 4G, H graph**

| **Fig 4G, H. ELISA** | | | | | |
| --- | --- | --- | --- | --- | --- |
| **IL-6** | **Mean 1** | **Mean 2** | **Mean Diff.** | **95.00% CI of diff.** | **Adjusted P Value** |
| Sham vs. 70 kPa | 1.313 | 3.854 | -2.542 | -5.806 to 0.7231 | 0.1394 |
| Sham vs. 80 kPa | 1.313 | 6.292 | -4.979 | -8.244 to -1.714 | 0.004 |
| Sham vs. 90 kPa | 1.313 | 7.188 | -5.875 | -9.140 to -2.610 | 0.0011 |
| **TNF-α** | **Mean 1** | **Mean 2** | **Mean Diff.** | **95.00% CI of diff.** | **Adjusted P Value** |
| Sham vs. 70 kPa | 393.8 | 400 | -6.25 | -304.6 to 292.1 | >0.9999 |
| Sham vs. 80 kPa | 393.8 | 625 | -231.3 | -529.6 to 67.09 | 0.1522 |
| Sham vs. 90 kPa | 393.8 | 787.5 | -393.8 | -692.1 to -95.41 | 0.0095 |

**S5 Table. The values used to build Fig 5A-D graph**

| **Fig 5A. BBB** | | | | | |
| --- | --- | --- | --- | --- | --- |
| **0 Weeks** | **Mean 1** | **Mean 2** | **Mean Diff.** | **95.00% CI of diff.** | **Adjusted P Value** |
| sham vs. 70 kpa | 21 | 21 | - | - | - |
| sham vs. 80 kpa | 21 | 21 | - | - | - |
| sham vs. 90 kpa | 21 | 21 | - | - | - |
| **1 Weeks** | **Mean 1** | **Mean 2** | **Mean Diff.** | **95.00% CI of diff.** | **Adjusted P Value** |
| sham vs. 70 kpa | 20.25 | 16.75 | 3.5 | 1.369 to 5.631 | 0.0128 |
| sham vs. 80 kpa | 20.25 | 14.5 | 5.75 | 4.684 to 6.816 | 0.0004 |
| sham vs. 90 kpa | 20.25 | 14.25 | 6 | 4.260 to 7.740 | 0.0015 |
| **2 Weeks** | **Mean 1** | **Mean 2** | **Mean Diff.** | **95.00% CI of diff.** | **Adjusted P Value** |
| sham vs. 70 kpa | 20.5 | 17.25 | 3.25 | 1.209 to 5.291 | 0.014 |
| sham vs. 80 kpa | 20.5 | 15.25 | 5.25 | 2.568 to 7.932 | 0.0078 |
| sham vs. 90 kpa | 20.5 | 15.25 | 5.25 | 3.209 to 7.291 | 0.0035 |
| **3 Weeks** | **Mean 1** | **Mean 2** | **Mean Diff.** | **95.00% CI of diff.** | **Adjusted P Value** |
| sham vs. 70 kpa | 20.75 | 17.5 | 3.25 | 1.209 to 5.291 | 0.014 |
| sham vs. 80 kpa | 20.75 | 16 | 4.75 | 2.709 to 6.791 | 0.0047 |
| sham vs. 90 kpa | 20.75 | 16.25 | 4.5 | 3.269 to 5.731 | 0.0012 |
| **4 Weeks** | **Mean 1** | **Mean 2** | **Mean Diff.** | **95.00% CI of diff.** | **Adjusted P Value** |
| sham vs. 70 kpa | 20.75 | 17.5 | 3.25 | 1.209 to 5.291 | 0.014 |
| sham vs. 80 kpa | 20.75 | 16 | 4.75 | 2.709 to 6.791 | 0.0047 |
| sham vs. 90 kpa | 20.75 | 16.25 | 4.5 | 3.269 to 5.731 | 0.0012 |

| **Fig 5B. Ladder** | | | | | |
| --- | --- | --- | --- | --- | --- |
| **0 Weeks** | **Mean 1** | **Mean 2** | **Mean Diff.** | **95.00% CI of diff.** | **Adjusted P Value** |
| sham vs. 70 kpa | 14.75 | 12.28 | 2.475 | -22.04 to 26.99 | 0.946 |
| sham vs. 80 kpa | 14.75 | 13.73 | 1.025 | -19.20 to 21.25 | 0.9919 |
| sham vs. 90 kpa | 14.75 | 16.03 | -1.283 | -34.05 to 31.48 | 0.9886 |
| **1 Weeks** | **Mean 1** | **Mean 2** | **Mean Diff.** | **95.00% CI of diff.** | **Adjusted P Value** |
| sham vs. 70 kpa | 14.73 | 25.5 | -10.78 | -27.93 to 6.377 | 0.1537 |
| sham vs. 80 kpa | 14.73 | 31.68 | -16.95 | -24.95 to -8.948 | 0.0062 |
| sham vs. 90 kpa | 14.73 | 31.73 | -17 | -32.86 to -1.144 | 0.0416 |
| **2 Weeks** | **Mean 1** | **Mean 2** | **Mean Diff.** | **95.00% CI of diff.** | **Adjusted P Value** |
| sham vs. 70 kpa | 13.08 | 25.33 | -12.25 | -31.10 to 6.605 | 0.1427 |
| sham vs. 80 kpa | 13.08 | 24.95 | -11.88 | -24.79 to 1.038 | 0.0621 |
| sham vs. 90 kpa | 13.08 | 25.4 | -12.33 | -29.42 to 4.766 | 0.1125 |
| **3 Weeks** | **Mean 1** | **Mean 2** | **Mean Diff.** | **95.00% CI of diff.** | **Adjusted P Value** |
| sham vs. 70 kpa | 5.85 | 18.4 | -12.55 | -33.15 to 8.055 | 0.1643 |
| sham vs. 80 kpa | 5.85 | 13.88 | -8.025 | -26.05 to 10.00 | 0.3014 |
| sham vs. 90 kpa | 5.85 | 12.6 | -6.75 | -27.99 to 14.49 | 0.4938 |
| **4 Weeks** | **Mean 1** | **Mean 2** | **Mean Diff.** | **95.00% CI of diff.** | **Adjusted P Value** |
| sham vs. 70 kpa | 4.467 | 6.833 | -2.367 | -12.75 to 8.017 | 0.2149 |
| sham vs. 80 kpa | 4.467 | 8.533 | -4.067 | -20.72 to 12.59 | 0.2011 |
| sham vs. 90 kpa | 4.467 | 5.267 | -0.8 | -27.43 to 25.83 | 0.8798 |

| **Fig 5C. Left latency** | | | | | |
| --- | --- | --- | --- | --- | --- |
| **0 Weeks** | **Mean 1** | **Mean 2** | **Mean Diff.** | **95.00% CI of diff.** | **Adjusted P Value** |
| sham vs. 70 kpa | 6.81 | 6.933 | -0.1225 | -3.789 to 3.544 | 0.9976 |
| sham vs. 80 kpa | 6.81 | 6.518 | 0.2925 | -4.275 to 4.860 | 0.9839 |
| sham vs. 90 kpa | 6.81 | 6.585 | 0.225 | -2.756 to 3.206 | 0.9747 |
| **1 Weeks** | **Mean 1** | **Mean 2** | **Mean Diff.** | **95.00% CI of diff.** | **Adjusted P Value** |
| sham vs. 70 kpa | 5.283 | 3.945 | 1.338 | -1.686 to 4.361 | 0.3046 |
| sham vs. 80 kpa | 5.283 | 2.968 | 2.315 | 0.2410 to 4.389 | 0.0374 |
| sham vs. 90 kpa | 5.283 | 2.838 | 2.445 | -0.4146 to 5.305 | 0.0746 |
| **2 Weeks** | **Mean 1** | **Mean 2** | **Mean Diff.** | **95.00% CI of diff.** | **Adjusted P Value** |
| sham vs. 70 kpa | 5.703 | 4.065 | 1.638 | -6.955 to 10.23 | 0.6083 |
| sham vs. 80 kpa | 5.703 | 2.708 | 2.996 | -0.8165 to 6.808 | 0.0786 |
| sham vs. 90 kpa | 5.703 | 2.518 | 3.186 | -0.7642 to 7.136 | 0.0749 |
| **3 Weeks** | **Mean 1** | **Mean 2** | **Mean Diff.** | **95.00% CI of diff.** | **Adjusted P Value** |
| sham vs. 70 kpa | 5.61 | 3.41 | 2.2 | -2.947 to 7.347 | 0.2255 |
| sham vs. 80 kpa | 5.61 | 2.343 | 3.268 | -1.117 to 7.652 | 0.0867 |
| sham vs. 90 kpa | 5.61 | 2.585 | 3.025 | -0.2010 to 6.251 | 0.0565 |
| **4 Weeks** | **Mean 1** | **Mean 2** | **Mean Diff.** | **95.00% CI of diff.** | **Adjusted P Value** |
| sham vs. 70 kpa | 4.733 | 3.32 | 1.413 | -3.785 to 6.612 | 0.4262 |
| sham vs. 80 kpa | 4.733 | 2.51 | 2.223 | 1.455 to 2.992 | 0.0062 |
| sham vs. 90 kpa | 4.733 | 2.3 | 2.433 | 0.6470 to 4.220 | 0.0275 |

| **Fig 5D. Right latency** | | | | | |
| --- | --- | --- | --- | --- | --- |
| **0 Weeks** | **Mean 1** | **Mean 2** | **Mean Diff.** | **95.00% CI of diff.** | **Adjusted P Value** |
| sham vs. 70 kpa | 7.058 | 5.793 | 1.265 | -3.315 to 5.845 | 0.5773 |
| sham vs. 80 kpa | 7.058 | 6.735 | 0.3225 | -2.117 to 2.762 | 0.8941 |
| sham vs. 90 kpa | 7.058 | 6.875 | 0.1825 | -3.402 to 3.767 | 0.9916 |
| **1 Weeks** | **Mean 1** | **Mean 2** | **Mean Diff.** | **95.00% CI of diff.** | **Adjusted P Value** |
| sham vs. 70 kpa | 6.04 | 4.08 | 1.96 | -4.861 to 8.781 | 0.3988 |
| sham vs. 80 kpa | 6.04 | 3.015 | 3.025 | -1.630 to 7.680 | 0.1428 |
| sham vs. 90 kpa | 6.04 | 2.81 | 3.23 | 0.3468 to 6.113 | 0.037 |
| **2 Weeks** | **Mean 1** | **Mean 2** | **Mean Diff.** | **95.00% CI of diff.** | **Adjusted P Value** |
| sham vs. 70 kpa | 5.413 | 3.287 | 2.127 | -19.15 to 23.40 | 0.4512 |
| sham vs. 80 kpa | 5.413 | 2.033 | 3.381 | 2.458 to 4.304 | 0.0039 |
| sham vs. 90 kpa | 5.413 | 2.265 | 3.148 | 0.7878 to 5.509 | 0.0287 |
| **3 Weeks** | **Mean 1** | **Mean 2** | **Mean Diff.** | **95.00% CI of diff.** | **Adjusted P Value** |
| sham vs. 70 kpa | 6.21 | 3.443 | 2.768 | -3.788 to 9.323 | 0.2299 |
| sham vs. 80 kpa | 6.21 | 2.815 | 3.395 | 2.621 to 4.169 | 0.0027 |
| sham vs. 90 kpa | 6.21 | 2.85 | 3.36 | 1.088 to 5.632 | 0.0235 |
| **4 Weeks** | **Mean 1** | **Mean 2** | **Mean Diff.** | **95.00% CI of diff.** | **Adjusted P Value** |
| sham vs. 70 kpa | 5.9 | 3.033 | 2.867 | -13.48 to 19.21 | 0.275 |
| sham vs. 80 kpa | 5.9 | 2.413 | 3.487 | -16.70 to 23.68 | 0.279 |
| sham vs. 90 kpa | 5.9 | 3.02 | 2.88 | -0.6864 to 6.446 | 0.0619 |
